# Electrical characterization and accelerated aging of amorphous silicon carbide implantable encapsulation

**DOI:** 10.1088/1741-2552/ae2956

**Published:** 2025-12-30

**Authors:** Christopher K Nguyen, Negar Geramifard, Yupeng Wu, Madhav Bhatt, Alexandra Joshi-Imre, Sandeep Negi, Stuart F Cogan

**Affiliations:** 1 Department of Bioengineering, The University of Texas at Dallas, Richardson, TX, United States of America; 2 Department of Materials Science and Engineering, The University of Texas at Dallas, Richardson, TX, United States of America; 3 Office of Research and Innovation, The University of Texas at Dallas, Richardson, TX, United States of America; 4 Blackrock Neurotech, Salt Lake City, UT, United States of America

**Keywords:** thin-film encapsulation, amorphous silicon carbide, microelectrode arrays, neural interfaces, leakage current, accelerated aging, electrical stress

## Abstract

*Objective*. Chronically implanted microelectrode arrays (MEAs) are used for stimulating and recording neural activity in research and clinical settings. However, their reliability can be compromised by insufficient encapsulation stability. Amorphous silicon carbide (a-SiC), a chemically stable and biocompatible material, has emerged as a potential thin-film encapsulation for MEAs. We aimed to evaluate thin-film a-SiC encapsulation using electrical-accelerated aging (EAA) and to demonstrate a methodology for obtaining acceleration factors for EAA by Weibull analysis. *Approach*. Interdigitated electrodes (IDEs) encapsulated with a-SiC were subjected to voltage cycling and stepped-voltage protocols to measure leakage currents in buffered saline at 37 °C. EAA employed incrementally increasing voltage biases over time to induce degradation and reveal failure mechanisms. *Main results*. IDEs exhibited a significant change in electrical behavior on exposure to saline, with failure initiating at specific voltages and accompanied by gas evolution at defect sites. Incremental voltage biasing revealed a capacitive-to-faradaic transition in leakage current response that was used as a failure criterion. *Significance*. Acceleration factors for voltage-driven accelerated aging of a-SiC thin-film encapsulation can be obtained by Weibull analysis using a mechanistic failure criterion. Breakdown occurs at processing-related defects in the a-SiC. This study demonstrates the use of EAA for evaluating failure in a-SiC thin-film encapsulation used in implantable MEAs. EAA is broadly applicable to thin-film MEAs and provides a highly relevant method of predicting implanted lifetimes of bioelectronics.

## 1. Introduction

Stable thin-film encapsulation is necessary for the long-term reliability of many types of microelectrode arrays (MEAs) used for neural stimulation and recording [1, 2]. Materials such as silicon oxide, silicon nitride, parylene-C, and silicone are used as encapsulation for MEAs [3]. However, encapsulation degradation, particularly in polymers, remains a limitation to the chronic performance of neural devices [4–6]. This study reports on the stability of amorphous silicon carbide (a-SiC) thin films assessed by electrical-accelerated aging (EAA) for MEA encapsulation. Deposited via plasma-enhanced chemical vapor deposition (PECVD) at moderate temperatures (<350 °C), a-SiC films are suitable for many substrates employed in implantable medical devices. The a-SiC is chemically stable with high electronic resistivity [7–10]. Previous studies have assessed the stability and barrier properties of PECVD a-SiC in saline environments [11, 12]; however, long-term stability tested under voltage-driven accelerated testing conditions, relevant to implantable devices, remains mostly unexplored [13, 14]. To address this gap, this study applies EAA using progressive-stress testing and introduces a capacitive-to-faradaic transition as an early failure marker for thin-film a-SiC encapsulation.

Lifetime assessments of medical devices commonly employ thermal-accelerated aging (TAA) [15]. In this passive method, samples are soaked in saline at elevated temperatures (>37 °C) to multiply aging by some factor that estimates lifetime based on chemical stability of constituent materials. However, elevated temperatures alone do not assess voltage-driven degradation mechanisms, critical for understanding failure in electrical thin-film devices. To address this, we applied voltage as an electrical stress to study the stability and failure mechanisms in thin-film PECVD a-SiC. EAA is a common method for evaluating the lifetime of semiconductors and electronics using voltage as an accelerating stress to test lifetime and reliability [16–18]. To improve efficiency and sensitivity, we employed a progressive-stress method for EAA, incrementally increasing voltage for rapid determination of encapsulation degradation mechanisms [19, 20]. This research investigates the electrical properties of a-SiC as an implantable encapsulation for neural stimulation MEAs. We first establish baseline encapsulation resistance, then characterize degradation under electrolyte exposure, and finally apply progressive-stress testing to evaluate failure initiation mechanisms and obtain acceleration factors.

## 2. Methods

### 2.1. Device fabrication

**Figure 1.**
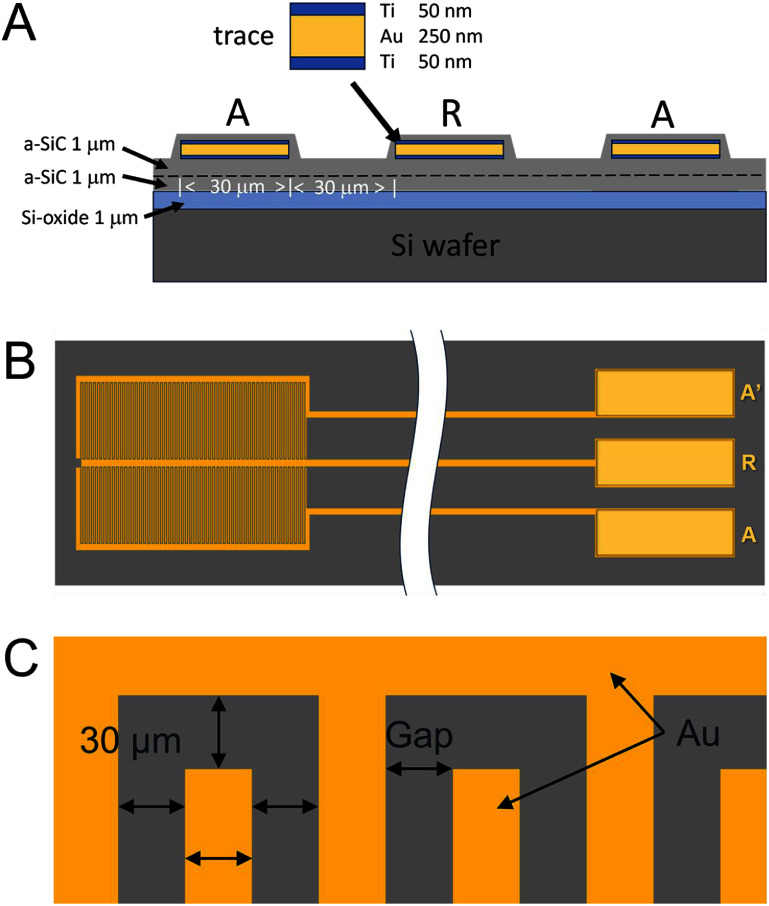
Interdigitated electrodes (IDEs) coated with a-SiC. (A) Cross-sectional schema of interdigitation showing individual layer thicknesses. (B) Active electrodes A and A’ flanking a common return electrode R; active electrode A’ was unused in this study. (C) Au interdigitation has the width and spacing of 30 *μ*m.

Planar interdigitated electrodes (IDEs) were used as a platform for measuring leakage current through a-SiC. IDEs are often employed to test the electrical properties of implantable materials [21]. IDEs were fabricated by photolithography on 100 mm Si (100) wafers. An isolation layer of 1 *μ*m SiO_2_ was thermally grown in a Tystar atmospheric furnace system (Tystar Corporation, USA). All patterning was made with lift-off resist LOR-5A and positive resist (MicroChemicals AZ® 1512) on a SÜSS MA6/BA6 Mask Aligner (SÜSS MicroTec, GE). The geometry of the interdigitated structure employs 30 *μ*m wide traces separated by a 30 *μ*m gap, as shown in figures 1(A) and (C). Prior to metallization, an initial 1 *μ*m layer of a-SiC film was deposited over the thermal SiO_2_ by PECVD using a Plasma-Therm Vision 310 Series system (Plasma-Therm, LLC, USA) with deposition parameters of 300 °C, gas pressure of 0.8 Torr, RF power of 200 W (power density of 0.36 W cm^−2^), and a total gas flow rate of 800 sccm (600 sccm of 2% SiH_4_ in Ar carrier gas, 36 sccm CH_4_, and 164 sccm Ar). The metallization layer comprised a tri-layer coating of Ti/Au/Ti (50/250/50 nm) deposited by electron-beam evaporation in a CHA Mark 50 system (CHA Industries, Inc, USA). A top a-SiC film was then deposited over the metallization to completely encapsulate the metal traces. Three top-layer a-SiC thicknesses were investigated. Films of 1- and 2 *μ*m to investigate leakage current pathways. For EAA, 500 nm a-SiC was used to emulate similar a-SiC thicknesses used in previous MEA studies [22, 23]. Bond pads to the metal IDEs were formed by opening vias through the top layer of a-SiC by reactive ion etching (RIE) using a Trion Sirus-T2 system (Trion Technology, USA) with etch parameters of 0.2 Torr, 200 W, and 10 sccm SF_6_. The RIE process also isolated individual IDEs by removing a-SiC between IDE structures on the wafer. Resists were stripped with AZ 5400T Photoresist Stripper. The samples were finally washed in acetone to remove resist residues and cleaned with IPA. The cross-sectional structure of the IDE and layer thickness is shown schematically in figure 1(A). An overview of IDE geometry is shown in figures 1(B) and (C): (B) image of IDE with traces to Au pads and (C) zoomed image of comb structures with gap and width dimensions. The design of the IDE structure results in a total finger surface areas of 4.7 mm^2^ for each comb electrode and a finger length of 2 mm. The gaps between combs A and R and between combs A’ and R are 30 *μ*m.

### 2.2. Leakage current measurements

**Figure 2.**
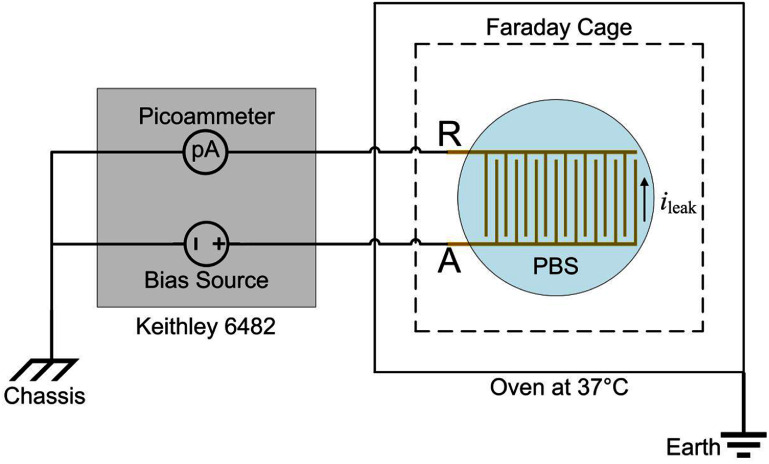
Schema of leakage measurement setup with the IDE immersed in PBS at 37 °C. The sample is sitting inside a floating Faraday cage, which is situated inside an oven. The ${V_{{\text{bias}}}}$ is applied between electrodes A and R and ${i_{{\text{leak}}}}$ measured with the picoammeter. Electrode A is the active electrode and ${V_{{\text{bias}}}}$ is reported with respect to Electrode R (return).

We used a Keithley Model 6482 dual-channel picoammeter/voltage source (Tektronix, Inc., USA) to test IDE samples by applying a DC voltage bias (${V_{{\text{bias}}}}$) and measuring leakage current (${i_{{\text{leak}}}}$) between IDE traces, as shown in figure 2. The current return path was established from active electrode A through the a-SC-encapsulated IDE to return electrode R, completing the circuit via the picoammeter’s internal ground return. The ${i_{{\text{leak}}}}$ measurements were used to quantify encapsulation integrity. For all measurements, the second flanking comb (A’ in figure 1(B)) was disconnected from the measuring circuit. All ${i_{{\text{leak}}}}$ measurements were taken at 37 °C. Initially, IDEs were measured in laboratory air to establish baseline properties of the a-SiC and to confirm the PECVD process was producing high-resistivity encapsulation. Subsequent measurements were made in in phosphate-buffered saline (PBS)—pH 7.1–7.3 and ionic conductivity ∼22 mS cm^−1^—representing for a more physiologically relevant environment. A script program was developed in MATLAB R2021b (MathWorks, Inc., USA) to automate the control of instruments and data collection [13]. The current was sampled at ${f_{\text{s}}}$ = 1 Hz. Current versus voltage (*I–V*) curves were generated by stepping ${V_{{\text{bias}}}}$ in increments over a pre-defined voltage range and measuring the steady-state ${i_{{\text{leak}}}}$ at each step. *I–V* measurements were used to characterize the electronic resistance of the encapsulation over the IDEs.

### 2.3. EAA

Electrical accelerated aging (EAA) typically employs large voltages (∼kV) [24, 25]. However, for implantable thin-film devices, particularly those immersed in ionically conductive body fluids, an operational ${V_{{\text{bias}}}}$ ⩽12 V has been used in thin-film neural devices for neural recording [26, 27] and simulation [28]. A ${V_{{\text{bias}}}}$ range of ±5 V was typically employed in this study to generate I–V curves for characterizing encapsulation resistance. In this study, ${V_{{\text{bias}}}}$ was extended to 30 V to deliberately accelerate aging and identify failure mechanisms.

There are two methods of applying voltage stress for EAA: constant-stress method, in which a fixed ${V_{{\text{bias}}}}$ is applied until failure is reached, and the progressive-stress method, where the ${V_{{\text{bias}}}}$ is incrementally increased over time [29]. In both cases, the time to encapsulation failure is the lifetime at the respective ${V_{{\text{bias}}}}$ when a pre-defined failure criterion is met. When ${i_{{\text{leak}}}}$ over time exhibits abrupt, transient changes, partial discharges (PDs) are inferred—partial breakdowns in the encapsulation [30, 31]. The accumulation of PDs degrades the film and gradually leads to full encapsulation failure [32]. Both constant-stress and progressive-stress methods follow the inverse power law (IPL) [33],

\begin{align*}L = \frac{1}{{K{V^{{n_{{\text{acc}}}}}}}},{ }{n_{{\text{acc}}}} &gt; 0.\end{align*}

In (1), $L$ is the lifetime, $V$ is the voltage stress level (${V_{{\text{bias}}}}$), $K$ is the inverse of maximum lifetime, and ${n_{{\text{acc}}}}$ is an acceleration constant of the material or device. From (1), we can write:

\begin{align*}\log \left( L \right) = - \log \left( K \right) - {n_{{\text{acc}}}}\log \left( V \right).\end{align*}

Equation (2) defines a linear relationship such that a plot of $\log \left( L \right)$ versus $\log \left( V \right)$ has slope -${n_{{\text{acc}}}}$ and $y$ -intercept -$\log \left( K \right)$. Using ${n_{{\text{acc}}}}$ determined from (2), an acceleration factor ${A_{\text{f}}}\left( V \right)$ can be calculated from two different ${V_{{\text{bias}}}}$ levels:

\begin{align*}{A_{\text{f}}}\left( V \right) = {\left( {\frac{{{V_{{\text{acc}}}}}}{{{V_{{\text{ref}}}}}}} \right)^{{n_{{\text{acc}}}}}},{ }{V_{{\text{ref}}}} \ne { }0.\end{align*}

**Figure 3.**
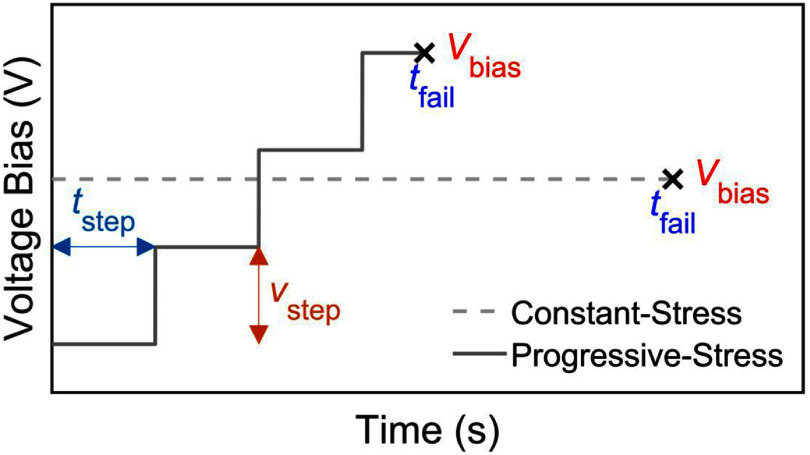
Conceptual examples of constant-stress and progressive-stress testing. Constant-stress method held at a voltage bias (${V_{{\text{bias}}}}$) reaching failure time (${t_{{\text{fail}}}}$). Progressive-stress reaching failure time (${t_{{\text{fail}}}}$) at a breakdown ${V_{{\text{bias}}}}$ with fixed voltage step (${v_{{\text{step}}}}$) and time step (${t_{{\text{step}}}}$),

From (3), a stress-level-dependent acceleration factor ${A_{\text{f}}}\left( V \right)$ is calculated at a ${V_{{\text{bias}}}}$ level that is beyond the operational condition to accelerate time (${V_{{\text{acc}}}}$) with respect to that operational or reference voltage (${V_{{\text{ref}}}}$), the operational voltage the encapsulation experiences. Although, the constant-stress method is an established approach for lifetime predictions, it can be experimentally time-consuming, because separate tests need many ${V_{{\text{bias}}}}$ levels. The efficiency of the progressive-stress method has been contrasted with the constant-stress in previous literature [19, 20], and these studies highlight that stepped-stress testing can significantly reduce the total test time while still yielding reliable lifetime estimates, making it particularly advantageous when resources or sample sizes are limited. Alternatively, the progressive-stress method involves a staircase function of increasing voltage at constant time and voltage steps until a failure time (${t_{{\text{fail}}}}$) and breakdown ${V_{{\text{bias}}}}$ are obtained [16, 34]. A comparison of constant ${V_{{\text{bias}}}}$ and staircase waveforms is shown in figure 3, with the staircase waveform defined by voltage bias increments (${v_{{\text{step}}}}$) and the time at each voltage bias (${t_{{\text{step}}}}$). The IPL can be applied to progressive-stress measurements using a quantity representing the total cumulative damage ($D$).

\begin{align*}L = \frac{D}{{{V^{{n_{{\text{acc}}}}}}}}\end{align*}

Equation (4) is a modified form of the IPL, where damage $D$ is the constant of interest rather than $K$, and can be written as:

\begin{align*}D = L \cdot {V^{{n_{{\text{acc}}}}}}\end{align*}

with $D$ determined by the summation,

\begin{align*}D = {t_{{\text{step}}}}\mathop \sum \limits_{i = 1}^{k - 1} \left[ {V_i^{{n_{{\text{acc}}}}}} \right] + {t_{{\text{final}}}} \cdot V_k^{{n_{{\text{acc}}}}}\end{align*}

where:

\begin{align*}{V_i} &amp;= {v_{{\text{step}}}} \cdot \left( {i - 1} \right)\end{align*}

\begin{align*}k &amp;=\left\lfloor\frac{L}{{{t_{{\text{step}}}}}}\right\rfloor + 1\end{align*}

\begin{align*}{t_{{\text{final}}}} &amp;= L - {t_{{\text{step}}}} \cdot \left( {k - 1} \right).\end{align*}

Equation (6) is the computation of $D$ from the total progressive stress for the designated ${t_{{\text{step}}}}$ and ${v_{{\text{step}}}}$ for $k$ steps such that the final step (${t_{{\text{final}}}}$) reflects the likely situation that failure is encountered part way through a given step [35, 36]. Equation (7) defines the stress level ${V_i}$ for $i$ th step, where $i$ = 1 corresponds to starting at 0 V (versus common return *R*, see figure 3). The values for $k$ step and ${t_{{\text{final}}}}$ are given by (8) and (9), respectively. Equations (6) and (7) are combined to give:

\begin{align*}D &amp;= {t_{{\text{step}}}}\mathop \sum \limits_{i = 1}^{k - 1} \left[ {{{\left( {{v_{{\text{step}}}} \cdot \left( {i - 1} \right)} \right)}^{{n_{{\text{acc}}}}}}} \right] \nonumber\\ &amp;\quad+\left( {L - {t_{{\text{step}}}} \cdot \left( {k - 1} \right)} \right) \cdot {\left( {{v_{{\text{step}}}} \cdot \left( {k - 1} \right)} \right)^{{n_{{\text{acc}}}}}}\end{align*}

$D$ in (5) relates to ${n_{{\text{acc}}}}$ and lifetime ($L$) at a voltage stress ($V$) through the IPL (6) giving the linear relationship:

\begin{align*}\log \left( D \right) = \log \left( L \right) + {n_{{\text{acc}}}}\log \left( V \right).\end{align*}

Two methods may be used to determine the ${n_{{\text{acc}}}}$ from progressive-stress measurements: (i) scanning the discrepancies (sum of combinational differences) of $\log \left( D \right)$ at different ${t_{{\text{step}}}}$ [13] and (ii) fitting the linear regression of $D$ at different ${t_{{\text{step}}}}$ [35]. The ${i_{{\text{leak}}}}$ was measured in response to ${V_{{\text{bias}}}}$ by stepping ${V_{{\text{bias}}}}$ (${v_{{\text{step}}}}$ = 0.1 V) up to 30 V at ${t_{{\text{step}}}}$ = 1, 2, and 5 min.

For the scanning method, (11) is used for algorithmically determining the best estimation of ${n_{{\text{acc}}}}$ (${\hat n_{{\text{acc}}}}$). The IPL relations can be defined for each ${t_{{\text{step}}}}$ as:

\begin{align*}\left\{ {\begin{array}{*{20}{c}} {\log \left( {{D_1}} \right) = \log \left( {{L_1}} \right) + {n_{{\text{acc}}}}\log \left( {{V_1}} \right)} \\ {\log \left( {{D_2}} \right) = \log \left( {{L_2}} \right) + {n_{{\text{acc}}}}\log \left( {{V_2}} \right)} \\ {\log \left( {{D_5}} \right) = \log \left( {{L_5}} \right) + {n_{{\text{acc}}}}\log \left( {{V_5}} \right)} \end{array}} \right.\end{align*}

where subscripts 1, 2, and 5 in (12) denote the different ${t_{{\text{step}}}}$ values used in this study. The discrepancy ($s$) calculated for the scanning method is given as:

\begin{align*}s = \frac{{\left| {\log \left( {{D_1}} \right) - \log \left( {{D_2}} \right){ }} \right| + \left| {\log \left( {{D_2}} \right) - \log \left( {{D_5}} \right){ }} \right| + \left| {\log \left( {{D_5}} \right) - \log \left( {{D_1}} \right){ }} \right|}}{{\sqrt {{n_{{\text{acc}}}} + 1} }}.\end{align*}

Equation (13) uses the sum of differences between $\log \left( D \right)$ of varying ${t_{{\text{step}}}}$ at a given ${n_{{\text{acc}}}}$. The ${n_{{\text{acc}}}}$ is substituted with values within a reasonable range of real numbers to find the best estimation (${\hat n_{{\text{acc}}}}$), given by:

\begin{align*}{\hat n_{{\text{acc}}}} = \min \left( s \right)\end{align*}

where (14) estimates ${\hat n_{{\text{acc}}}}$ at the minimum $s$.

For the fitting method, the linear regression is taken of different $D$ values over the respective $L$ values, notated as:

\begin{align*} X\left[ {{n_{{\text{acc}}}}} \right] &amp;= \left\{ {{L_1},{L_2},{L_5}} \right\} \nonumber\\ Y\left[ {{n_{{\text{acc}}}}} \right] &amp;= \left\{ {{D_1},{D_2},{D_5}} \right\}.\end{align*}

Equation (15) is the set $X$ of $L$ and the set $Y$ of $D$ with varying ${t_{{\text{step}}}}$ at a given ${n_{{\text{acc}}}}$. The linear regression is given by:

\begin{align*}y = {\beta _0} + {\beta _1} \cdot x\left( {{n_{{\text{acc}}}}} \right)\end{align*}

yielding ${\beta _0}$ as the $y$ -intercept and ${\beta _1}$ as the slope. The ${\hat n_{{\text{acc}}}}$ is determined at the slope closest to zero, given as:

\begin{align*}\mathop {\lim }\limits_{{\beta _1} \to 0} \left( {{n_{{\text{acc}}}}} \right) = {\hat n_{{\text{acc}}}}.\end{align*}

Regardless of the ${t_{{\text{step}}}}$ value, $D$ should be a constant, see (4).

### 2.4. Microscopy

Scanning electron microscopy (SEM) and energy-dispersive x-ray spectroscopy (EDS) were performed with a Zeiss Supra 40 SEM system (Carl Zeiss AG, GE). Optical microscopy (OM) was performed with a Zeiss Axio Imager.M2 mm system (Carl Zeiss AG, GE).

### 2.5. Statistical analysis

Statistical tests were performed in MATLAB R2021b, and most figures were generated in GraphPad Prism 10 (GraphPad Software Inc., USA). The Anderson-Darling test was used to determine normal and lognormal distributions, whereas the Lilliefors test was used to determine Weibull distribution. We used the $F$ -test for equality of variances and unpaired $t$ -tests to compare groups that exhibit lognormal distribution.

## 3. Results

### 3.1. a-SiC encapsulation

**Figure 4.**
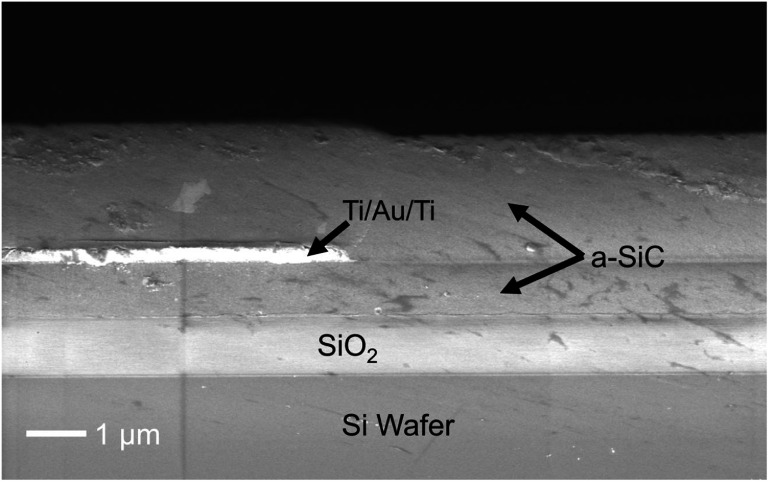
Cross-sectional SEM of cleaved IDE with 1 *μ*m bottom and 2 *μ*m top a-SiC layers.

The residual stress in the a-SiC films was compressive ($\hat \sigma $ = −80.5 ± 4.0 MPa), consistent with previous studies of PECVD a-SiC [8, 37]. Although PECVD is known to be prone to void formation due to poor step coverage, which can reduce reliability [38], no voids were observed in the a-SiC along the lateral edges of the IDE metallization combs. This was confirmed by examining several IDE samples from a single wafer. A representative cross-section of an IDE with 1- and 2 *μ*m a-SiC bottom and top layers, respectively, and 350 nm Ti/Au/Ti metallization is shown in figure 4.

### 3.2. Measurements in air

**Figure 5.**
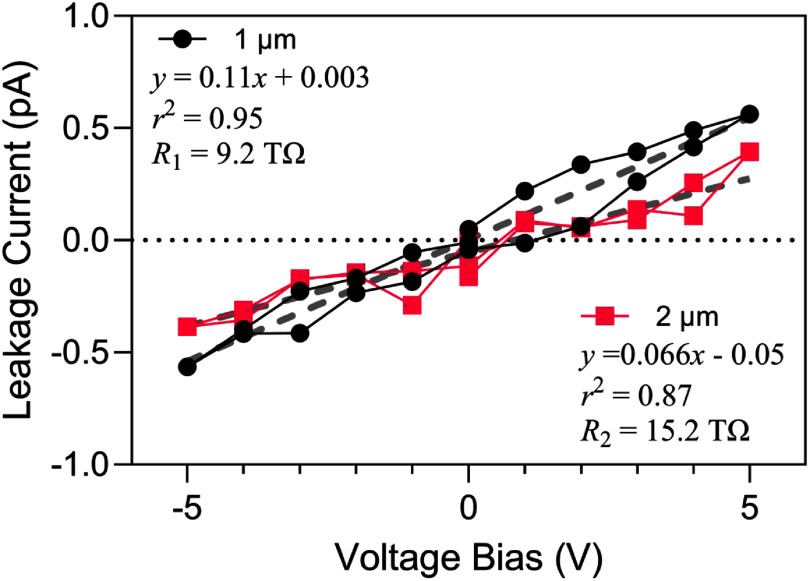
Representative resistance comparison of IDEs with 1 *μ*m and 2 *μ*m top-layer a-SiC layers. *I–V* curves include linear regression as dashed lines.

Initial *I–V* curves of IDE structures were obtained in laboratory air at 37 °C, providing electrical characterization of intrinsic a-SiC insulation without influence of electrolyte-induced effects. The *I–V* curves exhibited linear behavior across a ±5 V range, as shown in figure 5. Linear regression was used to determine the resistance of structures with 1- and 2 *μ*m a-SiC top layers, which result in 2- and 3 *μ*m total a-SiC thickness, respectively (a 3 *μ*m a-SiC IDE is shown in figure 4). Resistance measurements for thickness levels demonstrated lognormal distribution, which was confirmed by the Anderson-Darling test (1 *μ*m: $p$ = 0.27; 2 *μ*m: $p$ = 0.19). Table 1 lists the lognormal descriptive statistics for each thickness level, where there is equal variance between groups determined by $F$ -test ($p$ = 0.62) with standard deviation (SD) factor of 1.8. The resistance difference between 1- and 2 *μ*m top layers was statistically significant by unpaired $t$ -test ($p$ = 0.006). Increasing the a-SiC thickness from 1 to 2 *μ*m increases the a-SiC cross-sectional area, which reduces the bulk resistance of the a-SiC between combs. However, the observation in air that a ∼2-fold increase in resistance as the top a-SiC thickness is increased from 1 to 2 *μ*m, suggests that a pathway through the thickness of a-SiC over the interdigitated metallization, combined with surface conduction, dominates the current pathway. Surface conduction on a-SiC likely arises from absorption of water in the presence of ionic surface contamination in association with surface oxidation [21, 39].

**Table 1.** Lognormal descriptive statistics for each top-layer thickness.

Thickness (*μ*m)	Sample size	Geometric mean (TΩ)	SD factor	95% confidence interval (TΩ)
1	23	8.0	1.8	(6.1, 10.4)
2	19	15.6	1.8	(11.7, 20.9)

### 3.3. Measurements in PBS electrolyte

**Figure 6.**
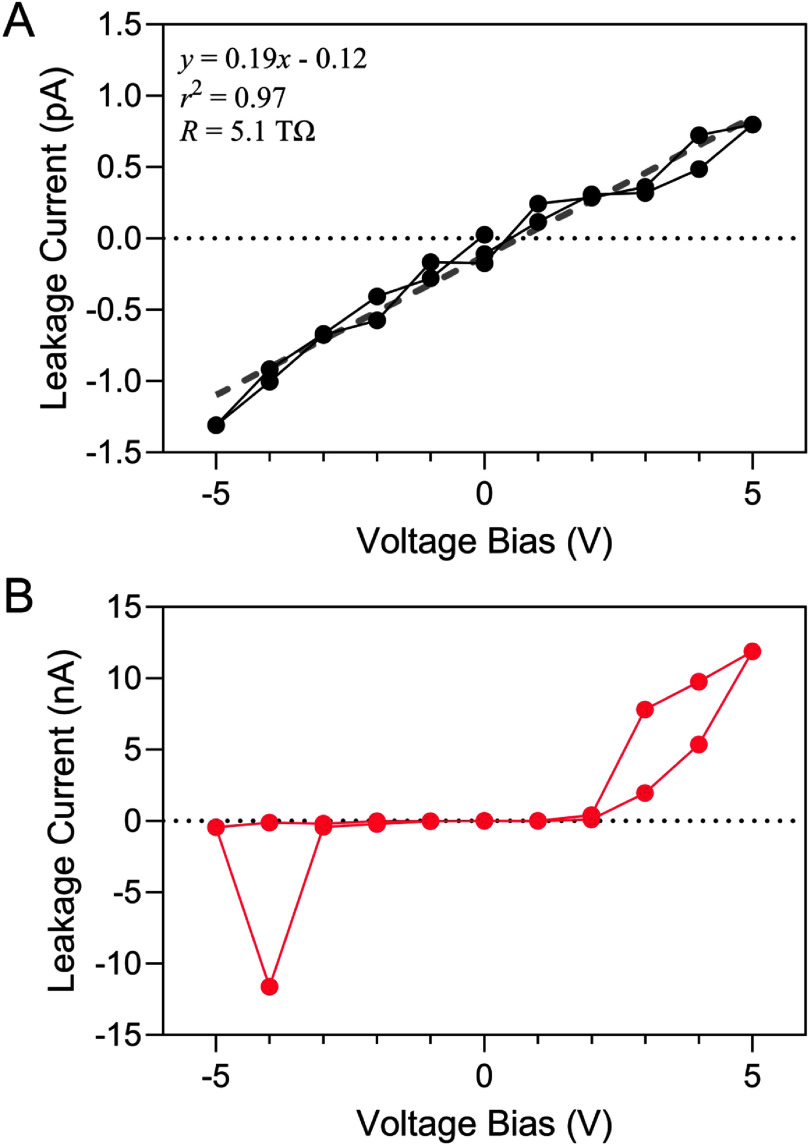
*I–V* curves of an IDE sample with 1 μm a-SiC top layer measured over ±5 V with a 1.0 V step size in: (A) air with linear regression indicating a 5.1 TΩ resistance and (B) in PBS showing large current excursions outside ±3 V.

After establishing baseline behavior in air, we evaluated leakage current in PBS to simulate physiological ionic conditions and observe electrolyte-induced degradation mechanisms. The *I–V* response of IDEs immersed in PBS is notably different from measurements in air. Representative *I–V* curves over ±5 V measured in air and after immersion in PBS are shown in figures 6(A) and (B), respectively. In PBS, the *I–V* curve shows a substantial increase in ${i_{{\text{leak}}}}$ at onset voltages of about 2 V on the positive scan and −3 V on the negative scan. These large current excursions are indicative of breakdown phenomena. At 2 V, the electric field across the total 2 *μ*m thickness of a-SiC, summed from the 1 *μ*m thickness on each IDE electrode, is 10 kV cm^−1^, notably less than typically reported for other dielectrics [40]. The onset of leakage current at 10 kV cm^−1^ does not correspond to the intrinsic voltage breakdown of a-SiC, but rather the influence of extrinsic deposition-related defects in the a-SiC film.

**Figure 7.**
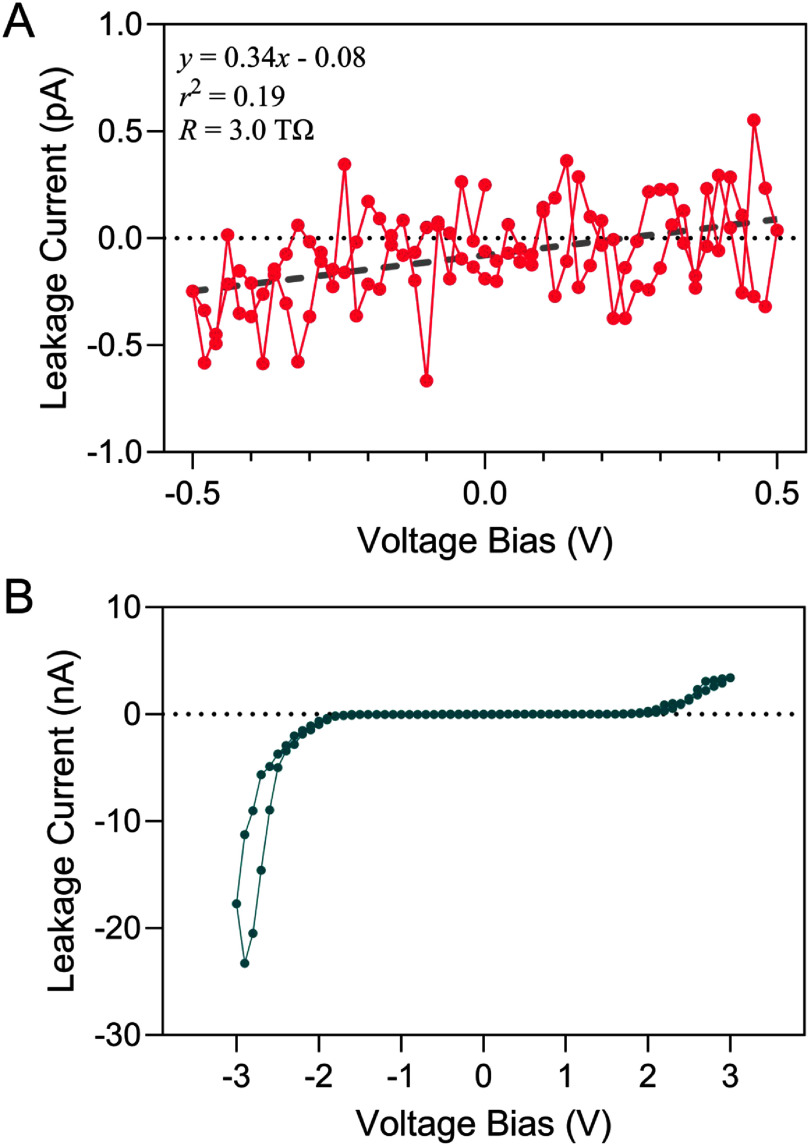
*I–V* curves of an IDE in PBS. Voltage range (A) between ±0.5 V with 0.01 V step size showed high-resistance similar to IDEs, and (B) between ±3 V with 0.1 V step size in which oxidation-reduction currents are observed.

**Figure 8.**
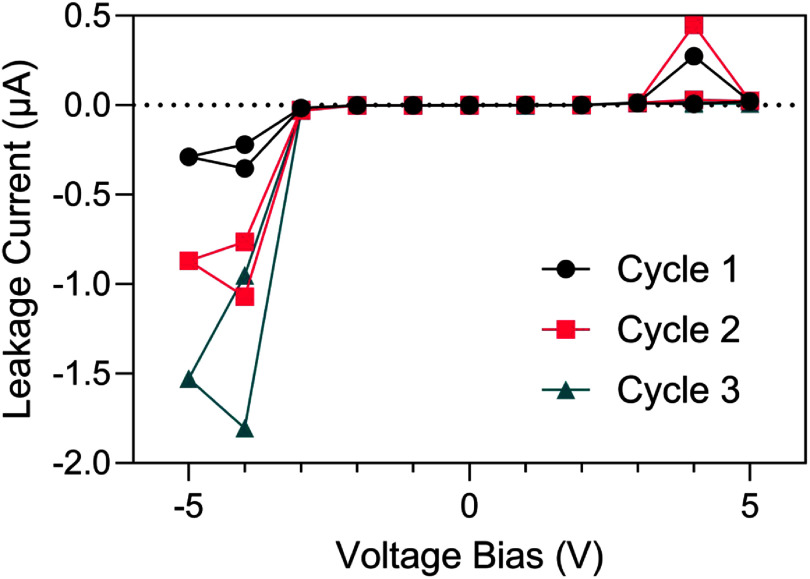
*I–V* response of an IDE sample with 1 *μ*m a-SiC top layer undergoing three consecutive *I–V* cycles between ±5 V at a 1.0 V step size. Each consecutive cycle induced increasingly larger leakage currents.

*I–V* measurements in PBS over smaller voltage ranges revealed features suggestive of electrochemical oxidation-reduction processes. As shown in figure 7(A), the interface leakage remained between ±2 pA for an *I–V* curve between ±0.5 V, consistent with measurements in air. However, increasing the range to ±3 V resulted in notable current onsets at approximately ±2 V (figure 7(B)) attributed to water electrolysis and incipient oxidation of the IDE metallization at sites of electrical breakdown or failure onset at the a-SiC. At larger voltages, the currents increased significantly and were accompanied by observable, localized breakdown of a-SiC passivation. This phenomenon illustrated in figure 8, shows progressively increasing currents after three consecutive cycles between ±5 V. The asymmetry in current between negative and positive limits is presumed to result from differences in the oxidation-reduction reaction rates at exposed metal at defects in the a-SiC. Large negative currents below −3 V are likely supported by water reduction, as evidenced by gas formation at localized sites on the IDE. In both figures 6(B) and 8, the decrease in current magnitude as the voltage is stepped from −4 V to −5 V is attributed to occlusion of exposed metal at defect sites by gas bubble formation.

**Figure 9.**
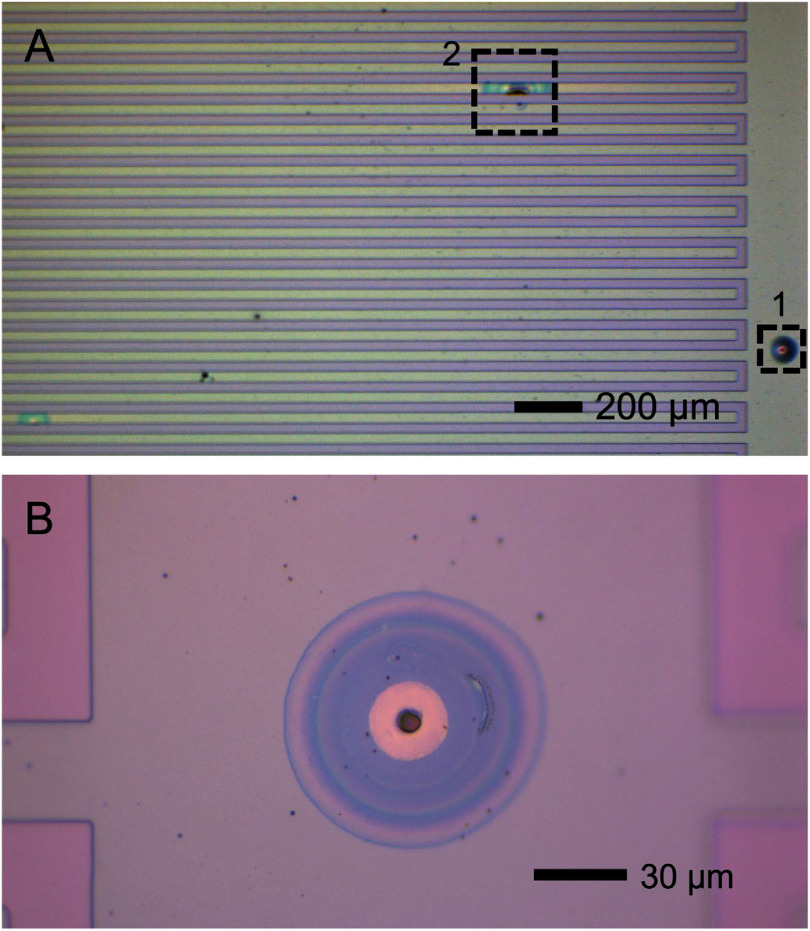
A sample with 1 *μ*m a-SiC thickness after three *I–V* cycles of ±5 V. (A) OM identification of two notable defect types. (B) Magnified defect 1.

**Figure 10.**
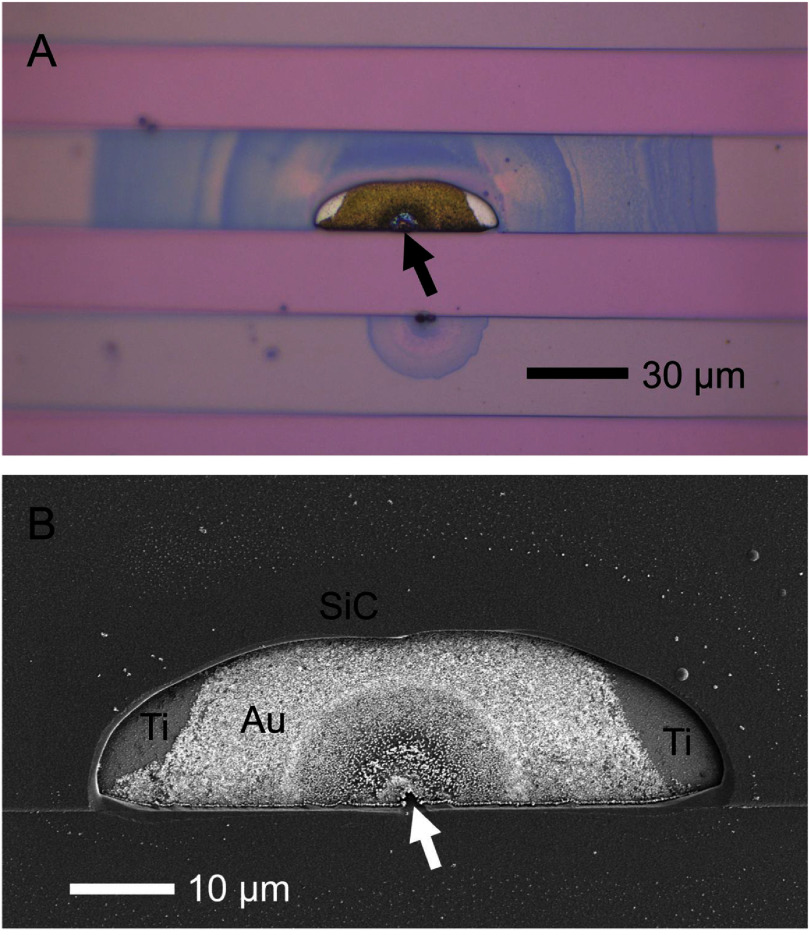
A sample with 1 *μ*m a-SiC thickness after three *I–V* cycles of ±5 V. (A) OM for defect 2. (B) SEM at for defect 2 with elements confirmed by EDS. Arrows denote location of defect origin.

After three *I–V* cycles of ±5 V, the response at voltage ranges beyond ±3 V varied depending on deposition-related defects, which appeared without a discernable pattern during each cycle. OM of example defects from this IDE are shown in figure 9. More detailed examination of the a-SiC encapsulation at breakdown sites by OM and SEM (figure 10), revealed localized breakdown and dissolution of the underlying metallization. These observations suggest breakdown of a-SiC at pre-existing defects in the a-SiC film. Possible defects include particulate contamination (defect 1 in figures 9(A) and (B)) and localized voids formed due to poor step coverage, potentially arising from process variability or coverage limitations at topographic features (defect 2 in figures 9(A) and 10), despite overall good step coverage observed in cross-section (figure 4). Although SEM of the cross section (figure 4) demonstrated good step coverage, the sparse occurrence of these defects, as suggested in figure 9(A), make it difficult to find defects in any one cross-section.

For example, Defect 1, observed in a-SiC over IDE metallization in figure 9(B), is characteristic of pinhole defects caused by particulate contamination that was dislodged during *I–V* cycling. A high-magnification image of defect 1 (figure 9(B)) shows a circular hole of ∼6 *μ*m diameter in the top a-SiC. defect 2, shown in figure 9(A), is located at the step coverage of a-SiC over the metallization in the interdigitation region. The OM in figure 10(A) reveals radial rings centered on the defect origin that suggest expanding encapsulation degradation and separation of the a-SiC top layer form the underlying metallization over a distance of >60 *μ*m from the defect origin. A smaller edge defect is also apparent on the adjacent comb and is presumably part of the current return path. SEM of the defect (figure 10(A)), reveals loss of a-SiC and exposure of underlying metallization. The degradation is likely caused by mechanical breakdown driven by dissolution of underlying metallization and gas bubble formation, initiated at the defect indicated by the arrow near the trace edge (figure 10(B)). The layer assignments in figure 10(B) were confirmed by EDS, which identified the exposed Au and Ti layers.

### 3.4. Progressive-stress method

**Figure 11.**
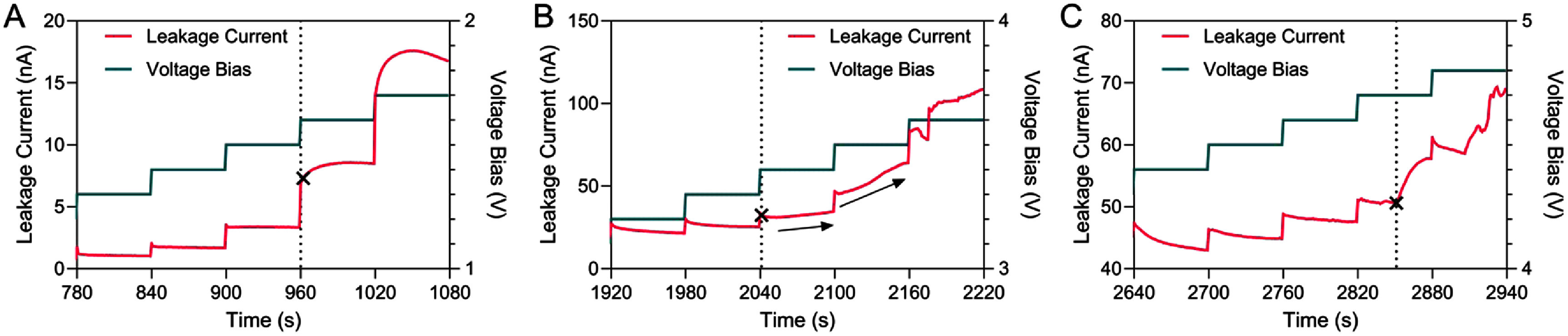
Progressive stress on three different IDEs samples with 500 nm a-SiC top layer in PBS showing failure onset at capacitive-to-faradaic transitions for ${t_{{\text{step}}}}$ = 1 min. (A) Simple change to faradaic reaction. (B) Accelerating current increase, indicated with arrows pointing upwards. (C) Onset of breakdown at mid-step. Cross symbol indicates the capacitive-to-faradaic transition.

While *I–V* measurements in PBS help reveal general trends in leakage and breakdown, they do not provide controlled conditions for lifetime prediction. Therefore, progressive-stress testing was used to systematically accelerate failure and quantify the onset of functional degradation. To determine failure time in progressive-stress testing employed a mechanistic failure criterion based on a transition from capacitive to faradaic current flow in response to a voltage step. This transition is illustrated in figure 11 for IDEs encapsulated with a 500 nm top layer of a-SiC. During initial voltage increments, the current response is capacitive, exhibiting an exponential decay to steady state over the duration of the time step (${t_{{\text{step}}}}$). At a higher ${V_{{\text{bias}}}}$, the current transitions from capacitive decay and begins to increase over the course of ${t_{{\text{step}}}}$.This behavior is presumed to be a faradaic response due to oxidation-reduction reactions occurring at exposed metallization, caused by localized a-SiC breakdown (as shown in figures 9 and 10 for IDEs with a 1 *μ*m a-SiC top layer). This capacitive-to-faradaic transition is described as the onset of functional failure for the a-SiC encapsulation, defining ${t_{{\text{fail}}}}$.

The change from capacitive to faradaic behavior may take different forms, as shown in figure 11. Regardless of the form, the capacitive-to-faradaic transition offers a mechanism-based threshold for identifying ${t_{{\text{fail}}}}$. The progressive-stress failures depicted in figure 11 were obtained using a ${t_{{\text{step}}}}$ of 1 min. This transition was detected using two criteria: (i) the step containing the transition must have a larger final value than the initial value, and (ii) the derivative analysis of the current must be increasing. The times to ${t_{{\text{fail}}}}$ for different ${t_{{\text{step}}}}$ durations are shown in table 2 and generally followed a lognormal distribution, as confirmed by the Anderson-Darling test. The $p$ -values for the distribution of ${t_{{\text{fail}}}}$, breakdown ${V_{{\text{bias}}}}$, and ${i_{{\text{leak}}}}$ are provided in table 2. The lognormal descriptive statistics are listed in table 3.

**Table 2.** Distribution $p$ -values for each time step.

Time step (min)	Sample size	Lognormal			Weibull
		Failure time	Breakdown voltage	Leakage current	Failure time
1	10	0.32	0.34	0.83	0.42
2	17	0.40	0.40	0.009	0.50
5	16	0.12	0.12	0.005	0.50

Lognormal distribution was determined by Anderson–Darling test.

Weibull distribution was determined by Lilliefors test.

**Table 3.** Onset of failure for each time step.

Time step (min)	Failure time (s)		Breakdown voltage (V)		Leakage current (nA)	
	Geometric mean	SD factor	Geometric mean	SD factor	Geometric mean	SD factor
1	1588.0	0.40	2.64	0.40	14.1	2.9
2	2323.4	0.47	1.94	0.47	8.7^a^	3.6
5	6134.9	0.41	2.04	0.41	18.0^a^	3.8

^a^ Does not pass log normality testing.

### 3.5. Weibull analysis

With the failure points identified from progressive-stress experiments, we applied Weibull statistical analysis to extract acceleration factors and assess the reliability of a-SiC encapsulation under voltage-driven aging conditions. The testing protocol was designed with a maximum ${V_{{\text{bias}}}}$ of 30 V. All failures occurred before reaching this 30 V limit, thus, not requiring right censoring [41]. The time to onset of failure (${t_{{\text{fail}}}}$) was confirmed to follow a Weibull distribution by a Lilliefors test (see $p$ -values in table 2). Observations of IDE failure at each ${t_{{\text{step}}}}$ were sorted from shortest to longest (table 4). Median ranks for the $y$ -values, representing the ‘plotting positions’ on a Weibull plot, were determined using Hazen’s formula [29], implemented by default in MATLAB’s statistics and machine learning toolbox,

\begin{align*}{F_T}\left( {{t_i}} \right) = \frac{{i - 0.5}}{n}\end{align*}

**Table 4.** Probability positions of failure time at different time step.

Index	1 min		2 min		5 min	
	Observation (s)	Position	Observation (s)	Position	Observation (s)	Position
1	961	0.05	1080	0.03	2700	0.03
2	1021	0.15	1200	0.09	3000	0.09
3	1080	0.25	1200	0.15	3600	0.16
4	1140	0.35	1440	0.21	3901	0.22
5	1380	0.45	1560	0.26	5401	0.28
6	1861	0.55	1680	0.32	6000	0.34
7	1921	0.65	2040	0.38	6300	0.41
8	2041	0.75	2160	0.44	6300	0.47
9	2851	0.85	2520	0.50	6600	0.53
10	2940	0.95	2640	0.56	7800	0.59
11			2880	0.62	7800	0.66
12			3120	0.68	8100	0.72
13			3720	0.74	9000	0.78
14			3840	0.79	9600	0.84
15			3960	0.85	9600	0.91
16			4200	0.91	10 200	0.97
17			4560	0.97		

Observation values are observed times to failure (${t_{{\text{fail}}}}$).

Position values are probability positions (${F_T}\left( {{t_i}} \right)$) on Weibull plot.

**Figure 12.**
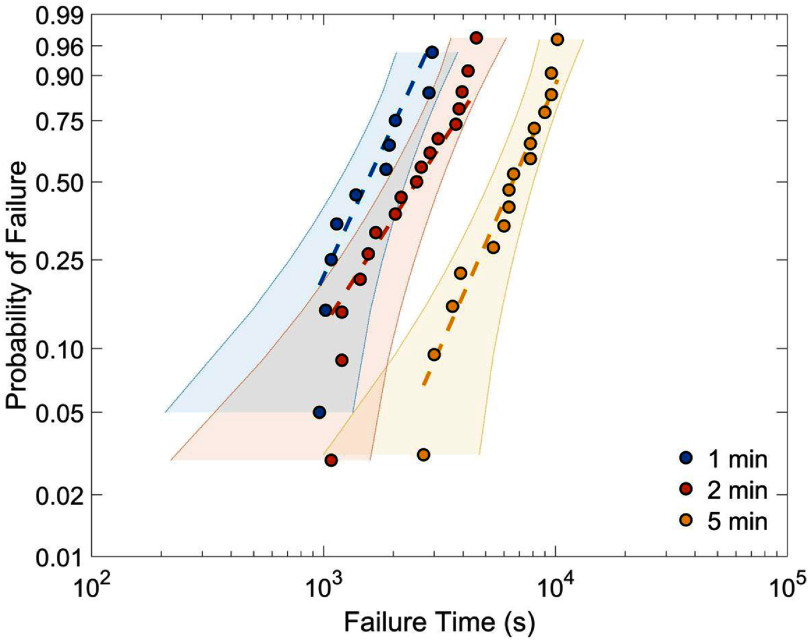
Weibull plots of different ${t_{{\text{step}}}}$. The progressive-stress parameters were ${v_{{\text{step}}}}$ = 0.1 V and ${t_{{\text{step}}}}$ = 1, 2, 5 min. Each data point is an observation of IDE failure. The shaded area indicates two-tailed 95% confidence bounds.

Equation (18) is the cumulative distribution function (CDF) of ${t_{{\text{fail}}}}$ as the probability of total number of sample failures, where $i$ is the index of the sorted set (from least to greatest) with sample size $n$. The probability position values from (19) are shown in table 4 and on the Weibull plots (figure 12) for each ${t_{{\text{step}}}}$ [42]. Two-tailed 95% confidence bounds for the Weibull plots were computed using beta-binomial bounds [43]. The overall CDF that describes the Weibull distribution is given by:

\begin{align*}{F_T}\left( t \right) = 1 - {e^{ - {{\left( {\frac{t}{a}} \right)}^b}}}\end{align*}

where $a$ is the scale parameter (characteristic life) and $b$ is the shape parameter (failure rate). These parameters were determined in MATLAB by fitting the data for each ${t_{{\text{step}}}}$ via maximum likelihood estimation. The resulting parameter values were subsequently used to estimate ${\hat n_{{\text{acc}}}}$. The scale parameter $a$ is the location where the bulk of the distribution lies and corresponds to the $L$ value in (12), for each ${t_{{\text{step}}}}$. The shape parameter $b$ is the ‘slope’ of failure probability versus failure time. The $b$ values in table 5 indicate wear-out failure ($b$ > 1) due to progressive degradation of the IDE devices.

**Table 5.** Weibull analysis of failure time and corresponding breakdown voltage.

		Times step		
		1 min	2 min	5 min
Scale, $a{ }$ (s)	Value	1942.8	2917.0	7412.8
	95% confidence interval	(1519.5, 2484.1)	(2389.2, 3561.4)	(5316.7, 8699.0)

Breakdown voltage (V)	Value	3.3	2.5	2.5
	95% confidence interval	(2.6, 4.2)	(2.0, 3.0)	(1.8, 2.9)

Shape, $b$	Value	2.7	2.5	3.2
	95% confidence interval	(1.7, 4.3)	(1.7, 3.7)	(2.1, 4.8)

**Figure 13.**
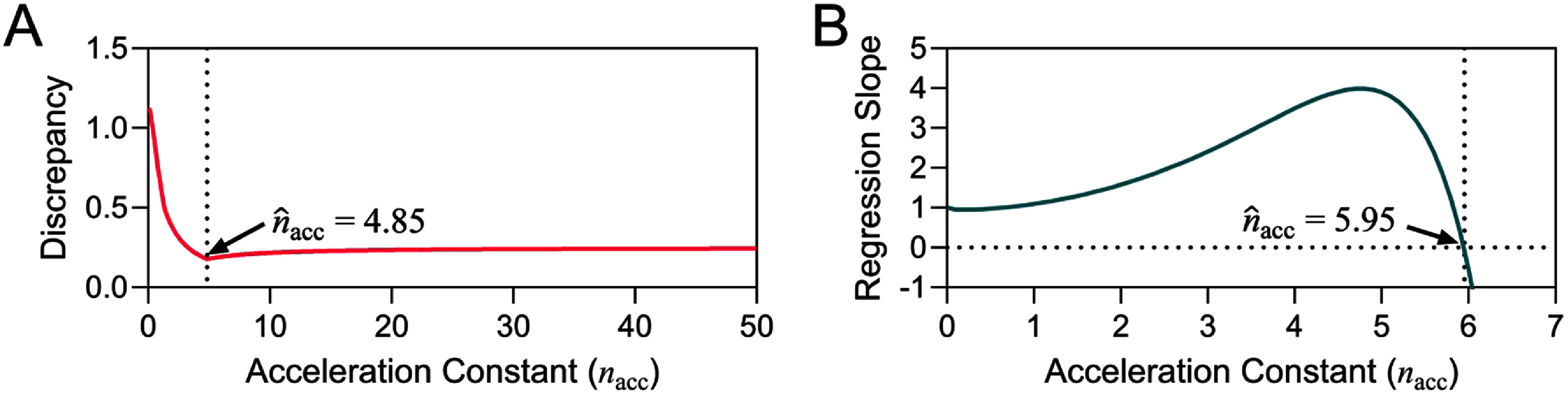
Graphical analysis a-SiC encapsulated IDEs that underwent progressive-stress testing. The progressive-stress data was analyzed and plotted by (A) the scanning method and (B) fitting method.

The ${\hat n_{{\text{acc}}}}$ was estimated by the scanning and fitting methods described in section 3.3. The scanning method evaluates the discrepancy $s$ (13) of different $\log \left( D \right)$ over all ${t_{{\text{step}}}}$, assuming ${n_{{\text{acc}}}}$ lies between 1 and 50 and selecting the point of smallest $s$, as shown in figure 13(A). The smallest $s$ was obtained at ${\hat n_{{\text{acc}}}}$ = 4.85. In contrast, the fitting method (16), shown in figure 13(B) yielded ${\hat n_{{\text{acc}}}}$ = 5.95. Whereas the two methods produced slightly different values, the differences impact the ${A_{\text{f}}}\left( V \right)$ significantly due to the IPL relationship (3). The range of ${A_{\text{f}}}\left( V \right)$ values allow an estimated lifetime range of the a-SiC encapsulated IDEs. For example, if the application uses a ${V_{{\text{ref}}}}$ of 5 V and ${V_{{\text{acc}}}}$ of 10 V, then ${A_{\text{f}}}\left( V \right)$ ranges from 28.8 to 61.8. Increasing the number of ${t_{{\text{step}}}}$ in future studies may refine and validate the ${\hat n_{{\text{acc}}}}$. It is important to note that ${A_{\text{f}}}\left( V \right)$ is specific to the a-SiC encapsulated IDEs tested in this study. For other devices and encapsulation materials, the ${\hat n_{{\text{acc}}}}$ would need to be independently determined. The progressive-stress method combined with Weibull analysis offers a systematic approach for estimating ${\hat n_{{\text{acc}}}}$ and characterizing the reliability of thin-film encapsulation.

## 4. Discussion

This study examined the long-term stability and failure mechanisms of a-SiC encapsulation under voltage-driven accelerated aging. A common method for determining failure during accelerated aging involves measuring ${i_{{\text{leak}}}}$ at a constant ${V_{{\text{bias}}}}$ over time until a pre-defined failure criterion, such as PD or total breakdown, is met [32]. Studies investigating the failure of encapsulation on IDEs might, for example, employ current thresholds of 1 nA [6] or 1 *μ*A [44]. In the present study, we used a capacitive-to-faradaic transition in ${i_{{\text{leak}}}}$ as a marker of failure initiation. To our knowledge, this study is the first to employ this transition as a failure marker for testing thin-film encapsulation on neural devices. The step-voltage conditions in this study were intended to demonstrate accelerated aging using the progressive-stress method. The a-SiC film was used as an example encapsulation material representative of coatings used for neural electrode arrays, not for integrated circuit (IC) packaging. In such applications, the electric field across the encapsulation is defined by the voltage applied at the electrode–tissue interface, typically <5 V, corresponding to the electric field of 2.5 × 10^4^ V cm^−1^ for a-SiC used in chronic implants [45]. These progressive-stress tests, therefore, represent intentional electrical overstress used to accelerate degradation and do not reflect normal operating conditions. The utility of the progressive-stress method for implantable encapsulation remains to be determined, but the present study provides a foundation for those wishing to explore it as a potentially more efficient and realistic approach to non-thermal accelerated aging.

We used a transition in ${i_{{\text{leak}}}}$ from capacitive decay to abruptly increasing, as a marker for the capacitive-to-faradaic transition that indicates the onset of encapsulation failure and also defines the cumulative damage (10) used in Weibull analysis. This strategy for identifying failure provides a more realistic indication of failure initiation compared to using a predefined or arbitrary ${i_{{\text{leak}}}}$ thresholds since faradaic reactions at the encapsulation are damaging to a device, as shown in figure 10. This transition allows us to detect failure initiation much earlier than waiting for a ${i_{{\text{leak}}}}$ to exceed a threshold. Traditional methods often miss the gradual degradation and partial breakdown occurring before the threshold is met, leading to delayed failure detection. This approach offers a mechanism-based failure criterion, improving the sensitivity of failure detection and providing earlier insights into device degradation. This method can be particularly useful in accelerated aging studies, where early failure detection can be critical in understanding long-term device stability.

The capacitive-to-faradaic behavior marks the onset of reduction or oxidation reactions at the metal traces, coincident with sufficient a-SiC encapsulation breakdown to allow water electrolysis at the metal and PBS electrolyte interface. Indeed, during *I–V* measurements exhibiting notable peaks exceeding ±3 V, bubble formation was observed on the IDE surface, indicating electrolysis and irreversible changes. The transient current response in the progressive-stress testing proceeding the capacitive-to-faradaic transition is mostly likely initiation of gas formation. The response could also be attributed to PDs—localized breakdown in the film—permitting more current flow, which is often observed in dielectric breakdown [46]. The capacitive-to-faradaic current transition is presumed to occur when the underlying metallization is exposed (figures 9 and 10). Device failure through trace corrosion and gas evolution will then proceed resulting in mechanical failure of the encapsulation as well as dissolution of trace metal. For stimulation applications, current shunting at failure sites can reduce the effective stimulation charge delivery to the target tissue and introduce an uncertain electric field distribution that might produce unanticipated neural recruitment.

Previous studies have reported very low ${i_{{\text{leak}}}}$ through a-SiC, <10 pA over a ±5 V range [11]. However, in the latter study, the a-SiC was deposited directly onto a thermal oxide layer, avoiding edge-coverage defects at the underlying, patterned metallization. We observed failure at defects associated with particulate contamination (figure 9) and at edges of the metal traces (figure 10). These failures occur at processing-induced defects and, while not intrinsic to the a-SiC, reflect the challenges associated with PECVD of inorganic encapsulation [47, 48]. Additionally, the extrinsic defect densities in thin-film fabrication depend on specific processing tools and facilities, which introduce uncertainty in lifetime predictions. The observed differences in the transition voltages across different samples (figure 11) can be attributed to multiple factors, likely arising from variability in extrinsic defects such as particulate contamination or voids in the a-SiC film at steps of coated metallization edges. These variability inherent in these defects is presumed to underline the wide range of transition voltages observed between samples and underscore the importance of controlling processing conditions to minimize defects. As noted previously, progressive-stress testing is device-specific and may require establishing the ${\hat n_{{\text{acc}}}}$ for each unique device structure. For stimulation electrodes, driving voltages of 1–5 V are often encountered [22, 37], resulting in an electric field of 10–50 kV cm^−1^ for a 500 nm a-SiC film thickness, which is comparable with that employed in the present progressive-stress study. If higher voltages, and consequently higher electrical fields, are required to obtain acceleration factors, consideration will need to be given to the introduction of failure mechanisms in the encapsulation that would not be realistically encountered with implanted neural stimulation devices.

Additionally, while this study defined failure based on a capacitive-to-faradaic transition over each ${t_{{\text{step}}}}$, we acknowledge that abrupt changes in the initial polarization (i.e. access resistance) observed at the onset of the ${V_{{\text{bias}}}}$ could reflect early encapsulation degradation or localized geometry changes. Future studies could investigate polarization shifts at the ${t_{{\text{step}}}}$ as an earlier indicator of failure onset. Most neural devices with implanted ICs employ a hermetic metal or ceramic enclosure and, in more limited cases, polymer encapsulation over conventional thin-film passivation for device encapsulation [49]. More recent devices can also employ IC elements implanted within the parenchyma of the target tissue [50, 51]. Such devices generally rely on the stability of inorganic passivation layers which are often silicon oxynitride or multilayer oxide and nitride coatings [47]. Of interest would be the determination of voltage-driven accelerated testing parameters for these coatings, including how they might perform as multi-layer passivation in combination with a-SiC.

In the progressive-stress study, we obtained ${A_{\text{f}}}\left( V \right)$ for 500 nm thick a-SiC deposited over patterned 350 nm thick metallization. This thickness of a-SiC was chosen to match that used in the development of flexible a-SiC/polyimide ribbon cables [23] as well as being the nominal thickness of a-SiC being studied as encapsulation for the shafts of Utah electrode arrays (UEAs) [22, 52]. Thicker a-SiC coatings, although still susceptible to defect-driven failure, shown in figures 9 and 10 for a 1 *μ*m a-SiC coating, are also likely less prone to failure [45]. As observed from *I–V* curves of IDEs in air with different a-SiC thicknesses, the ${i_{{\text{leak}}}}$ path between IDE electrode combs is primarily through the thickness of the a-SiC to the device surface. Therefore, larger breakdown ${V_{{\text{bias}}}}$ values are expected for thicker a-SiC before failure is initiated. Whereas the progressive-stress Weibull study focused on 500-nm thick a-SiC employed previously for encapsulating Utah-style electrodes arrays [22] and in a-SiC/polyimide ribbon cables [23], future work evaluating thicker films is warranted to explore how increased thickness mitigates defect-driven failure and extends device lifetime under EAA. One approach to extending the lifetime of a-SiC encapsulation is the use of polymer overlayers such as those employed in polyimide/a-SiC ribbon cables. Characterization of the polyimide/a-SiC structures by the progressive-stress method and possible lifetime enhancement obtained with a polymer overlayer would be useful future studies.

The selection of ${t_{{\text{step}}}}$ depends on the intended application environment. Longer ${t_{{\text{step}}}}$ values are more representative of continuous DC voltage stress as encountered by implanted ICs. In contrast, neural stimulation typically involves short pulse widths, suggesting that ${V_{{\text{bias}}}}$ levels may be needed to induce comparable damage at shorter ${t_{{\text{step}}}}$ values. We anticipate that the ${V_{{\text{bias}}}}$ at the onset of failure will increase with shorter ${t_{{\text{step}}}}$ and thicker encapsulation compared to the 500 nm a-SiC used in this study. For example, the minimum ${t_{{\text{step}}}}$ of 1 min (table 5) is substantially longer than pulse durations typically encountered in neural stimulation, which can range from 25 *μ*s to 1 ms [53, 54]. At shorter ${t_{{\text{step}}}}$, there is reduced time for capacitive charging, faradaic reactions, and defect growth to occur at each voltage increment, requiring a higher ${V_{{\text{bias}}}}$ to accumulate sufficient damage to initiate encapsulation failure. Since encapsulation failure may involve an incubation period at breakdown ${V_{{\text{bias}}}}$ before degradation develops, as observed in passivation breakdown of metal alloys [55, 56], significantly higher ${V_{{\text{bias}}}}$ is expected at ${t_{{\text{step}}}}$ on the order of stimulation pulse widths. A previous study of UEAs with a-SiC encapsulation, varying from 140 nm to 1.78 *μ*m sin thickness, demonstrated chronic stability for neural recording and stimulation in rat cortex under limited current pulsing with driving voltages <1.5 V. Although thinner a-SiC coatings are more vulnerable to defect-driven failure, their long-term stability under low driving voltages highlights the importance of considering application-specific stress conditions. Unlike the continuously applied, stepped ${V_{{\text{bias}}}}$ employed here to induce a-SiC degradation, clinical neuromodulation devices typically use pulsatile waveforms. Future studies should explore stepped-voltage waveforms that better represent clinical stimulation waveforms and investigate the performance and failure mechanisms of thicker a-SiC coatings.

## 5. Conclusion

A method was developed to quantify the impact of defects within thin-film encapsulation electrically stressed in an inorganic buffered electrolyte. By using the progressive-stress method and a capacitive-to-faradaic current response as the failure criterion, acceleration factors for EAA of a-SiC-coated IDE test structures were determined. The approach proved to be efficient, requiring only a few hours to obtain failure data under the specified test conditions. Failures in the a-SiC encapsulation were primarily associated with particulate contamination and defects at steps at the coated edges of the IDE metallization. While eliminating defect generation during fabrication is challenging, the reliability of thin-film encapsulation for bioelectrical interfaces can be realistically characterized. The findings underscore the utility of EAA in understanding the failure mechanisms of thin-film encapsulation and highlight the potential of EAA to guide the development of chronically stable thin-film bioelectronic implants.

## Data Availability

The data supporting the findings of this study are available from the authors upon reasonable request.
